# Orthoform

**Published:** 1898-11

**Authors:** T. Klaussner


### SELECTIONS.

### Orthoform.

BY PROFESSOR T. KLAUSSNER.

During the past month the new local anesthetic, orthoform, described by Prof. Einhorn and Dr. Heinz in No. 34 of the *Muenchener Medicin. Wochenschrift*, has been tried extensively in the Surgical Department of the Royal Polyclinic. This is not the time nor place for a detailed account of our investigations. It may be of general interest however, to report in brief the results obtained.

Although our experience with orthoform is still comparatively slight, we do not hesitate to predict that this new remedy is destined to occupy in the future a prominent place in the estimation of surgeons. Its most valuable properties are its slow solubility, which it shares with iodoform, and its absolute innocuity. Its anesthetic effect is developed whenever it comes into contact with an *open* wound, i. e., the exposed ends of the nerve filaments; it is inert in contact with normal epidermis or mucous membranes

The following conclusions can be drawn from the numerous observations of its use on open wounds, burns of the second and third degree, on syphilitic, varicose and carcinomatous ulcers, dental caries, etc.

(1) The anesthesia begins three to five minutes after the application of orthoform, in powder form or embodied in a ten to twenty percent ointment.

(2) The anesthesia lasts on an average thirty hours, in many cases even three or four days; in one case the effect disappeared within two hours, the powder having been washed away by the profuse discharge.

(3) Its influence in diminishing the discharge from wounds was evident, a quality which was found especially advantageous in making Thiersch's graftings. In a case of inoperable cancer of the buccal mucous membrane of the mouth it controlled the very trying excessive salivation to a striking degree.

(4) The freedom from toxic properties is vouched for by the fact, that in one case of cancer 60.0 grammes (℥ ii) were applied in one week without any disagreeable effect.

(5) No special attempt was made to test the *antiseptic properties* of the remedy on patients; however, no injurious influence on wounds in this respect was ever observed; it never caused suppuration, and suppurating wounds dried rapidly under its use.

In almost all our cases the base, orthoform itself, was used. The muriate of orthoform anesthetizes equally well, but creates intense pain at first upon its application, so that it will hardly become useful in surgery.

Dr. Wallenberger will soon publish a detailed account of our observations, with a report of cases.—*Muenchener Medicinische Wochenschrift*, No. 46, 1897.

